# A New Prospect in Road Traffic Energy Harvesting Using Lead-Free Piezoceramics

**DOI:** 10.3390/ma12223725

**Published:** 2019-11-11

**Authors:** Manuel Vázquez-Rodríguez, Francisco J. Jiménez, Lorena Pardo, Pilar Ochoa, Amador M. González, José de Frutos

**Affiliations:** 1 DTE-ETSIST, Universidad Politécnica de Madrid, 28031 Madrid, Spain; 2 CEMDATIC-POEMMA R & D Group, Universidad Politécnica de Madrid, 28040 Madrid, Spain; 3 Departamento de Electrónica Física, Ingeniería Eléctrica y Física Aplicada, Universidad Politécnica de Madrid, 28031 Madrid, Spain; 4 Instituto de Ciencia de Materiales de Madrid (ICMM), Consejo Superior de investigaciones Científicas (CSIC), C/Sor Juana Inés de la Cruz, 3. Cantoblanco 28049 Madrid, Spain

**Keywords:** piezoelectric ceramics, lead-free piezoceramics, energy harvesting, virtual instrument

## Abstract

In this paper, a new prospect using lead-free piezoelectric ceramics is presented in order to determine their behavior in piezoelectric-based road traffic energy harvesting applications. This paper will describe the low-cost and fully programmable novel test bench developed. The test bench includes a traffic simulator and acquires the electrical signals of the piezoelectric materials and the energy harvested when stress is produced by analogous mechanical stimuli to road traffic effects. This new computer-controlled laboratory instrument is able to obtain the active electrical model of the piezoelectric materials and the generalized linear equivalent electrical model of the energy storage and harvesting circuits in an accurate and automatized empirical process. The models are originals and predict the extracted maximum power. The methodology presented allows the use of only two load resistor values to empirically verify the value of the output impedance of the harvester previously determined by simulations. This parameter is unknown a priori and is very relevant for optimizing the energy harvesting process based on maximum power point algorithms. The relative error achieved between the theoretical analysis by applying the models and the practical tests with real harvesting systems is under 3%. The environmental concerns are explored, highlighting the main differences between lead-containing (lead zirconate titanate, PZT) and lead-free commercial piezoelectric ceramics in road traffic energy harvesting applications.

## 1. Introduction

Nowadays, climate change is one of the most extended concerned topics worldwide. Classical electrical energy generation models have opened toward clean energies, reducing their carbon footprint by gradually increasing the power produced in hydroelectric, wind, and solar power plants. However, this trend is still far from achieving that as most of the electrical production comes from energy with low CO_2_ emission to the atmosphere. For context, the 2018 annual report [1] about the Spanish electrical system shows that 19.8% was wind production, 13.8% was hydraulic, and 4.8% was solar (thermal, 1.8%, and photovoltaic, 3%).

Other subjects related to the environmental concerns are the reduction of harmful chemical waste, i.e., electrochemical accumulators or other electronic components that use lead (Pb) in their composition [2].

New techniques have been developed in micro-renewable energy generation, namely energy harvesting applications. Energy harvesting can be defined as electrical energy generation from natural and clean primary energy sources or from human activity to power electronic devices of low consumption. Some examples are wearable electronics, IoT (Internet of Things) devices, or wireless sensor networks. The source energies [3] are the well-known wind, solar, and mechanical energy from vibration, stress, or impacts generated from ambient or in residential or industrial human activities. Other primary energy sources are thermal energy and the RF (radio frequency) spectrum produced by human broadcast and telecommunication networks.

Applications in piezoelectric energy harvesting have been published since the beginning of the 21st century. The mechanical source is vibrational and the prevalent shape of the electromechanical transducer is the cantilever. Several enhancements were built-in such as magnetic elements, springs, L-shapes, and connections between them [4] to broaden the frequencies where maximum power generation is achieved.

The framework of the applied research presented in this article is electrical energy generation using ceramic piezoelectric transducers that optimizing the energy conversion from mechanical road traffic stimuli. A comparison is done between the behavior of lead-containing lead zirconate titanate (PZT) and lead-free commercial piezoelectric ceramics.

Table 1 shows a review of road traffic piezoelectric energy harvesting publications from 2010.

The main things lacking that has been appreciated in the previous review are summarized in the following. There is a reduced number of piezoelectric harvesters in roadway installations; instead, laboratory tests mostly apply uniaxial stress by means of Universal Test Machine (UTM) equipment. There is a low number of models of piezoelectric elements in road traffic environments. The influence of the instruments in the experimental measurements is generally not considered. The scalability of the power generated by harvesters is often not demonstrated.

The 2014 report [18] for the California Energy Commission estimated a high cost, at $600,000–$1,000,000, of a demonstration project that included laboratory, acceleration, and field tests.

This paper will describe the low-cost original and fully programmable instrument developed by some of the authors at the Universidad Politécnica de Madrid [19]. This test bench is able to obtain accurate models of piezoelectric-based energy harvesters and carry out the accelerated tests in a much more economically affordable way. The test bench includes a traffic simulator and acquires the electrical signals of the piezoelectric materials and the energy harvested when the stress is produced by mechanical stimuli, analogous to the road traffic effect.

The parameters of those models, as well as the harvested power, will be empirically verified by performing a reduced set of practical tests.

Finally, the main differences in energy harvesting applications between PZT and lead-free commercial piezoelectric ceramics will be highlighted.

## 2. The New Piezoelectric Characterization System

**Figure 1:**
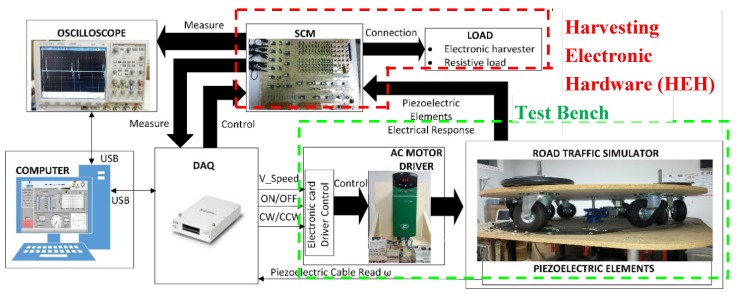
Piezoelectric characterization system block diagram.

Figure 1 shows a block diagram of the complete harvesting and piezoelectric test system. The test bench is made up of a Road Traffic Simulator driven by an AC geared motor. The angular speed *ω* (expressed in rpm) of the upper rotating platform shaft is fully programmable. The mechanical topology of this platform is built in an open way. Their wheels may be disposed in several locations to configure the angle between the simulated axes of the vehicles *β* (°). The static platform, below the rotating upper platform, includes, in the track way, the piezoelectric devices under test (PDUTs). Equation (1) calculates the simulated speed *v* (km/h) of the tests for each vehicle type. The data acquisition card (DAQ) sends the control signals to the driver control electronic card, which commands the AC motor driver.

(1) $$v=\frac{21.6\cdot b\cdot \omega }{\beta }.$$

The simulated speed in the test bench for a sedan-style car, which has a wheelbase, *b* (m), of 2.64 m, is between 14 km/h (8 mph) and 180 km/h (112 mph), as a maximum value for laboratory test purposes only.

**Figure 2:**
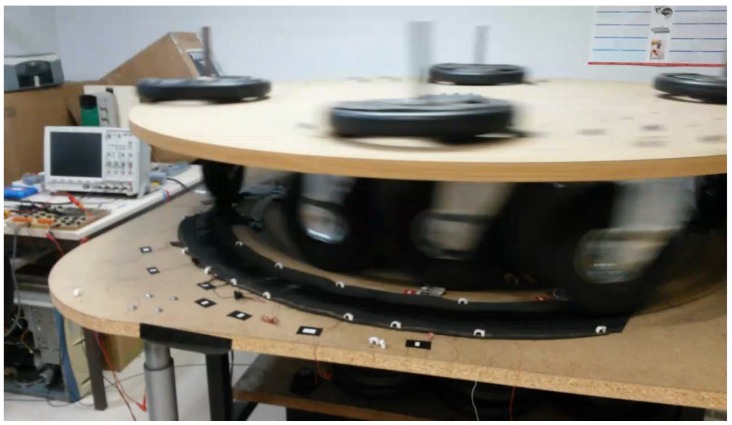
Piezoelectric characterization system in action.

A picture of the system performing the laboratory test is shown in Figure 2.

**Figure 3:**
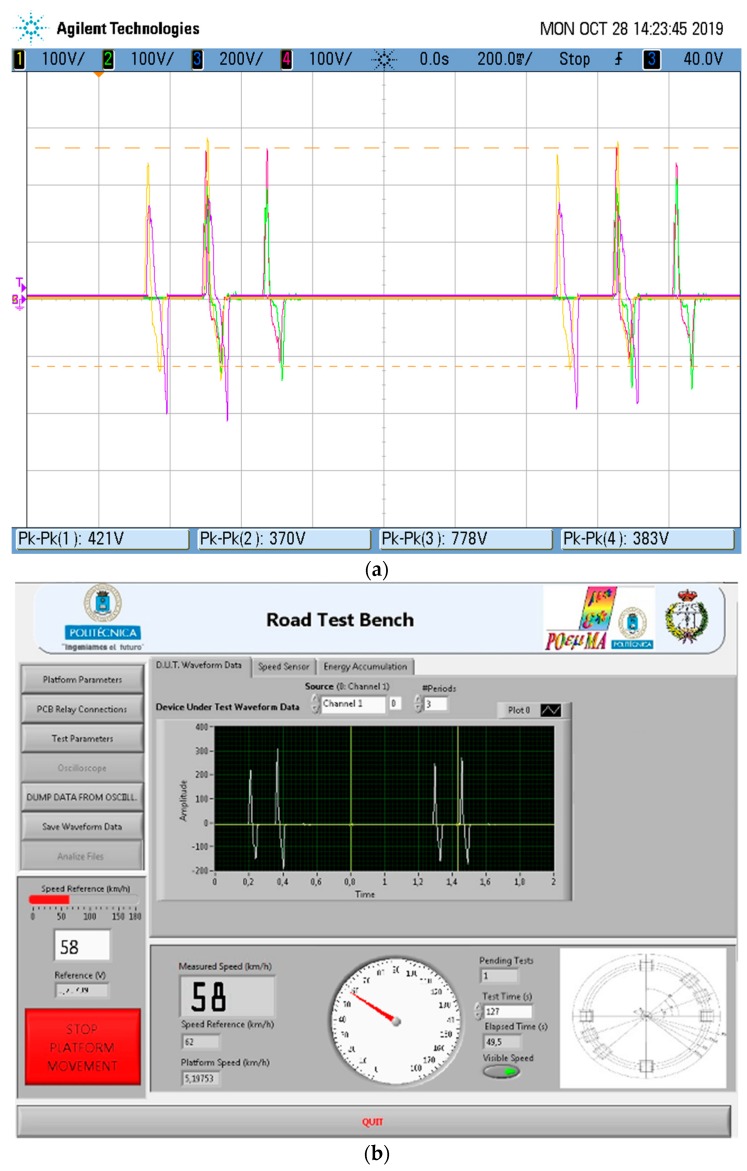
(**a**) Piezoelectric electrical signals from four lead-containing lead zirconate titanate (PZT) piezoelectric devices under test (PDUTs). (**b**) Software interface acquiring one channel of electrical PDUT response to obtain the active electrical model.

The harvesting electronic hardware (HEH) in Figure 1 performs the automatized electrical measurement. The relay-based switched circuit’s matrix (SCM) is electronically controlled by the DAQ. The SCM can control up to six PDUTs. The first routing stage of this matrix connects the PDUTs to a USB-controlled oscilloscope or to the selected diode rectifier topologies. The SCM second routing stage selects the rectifier topology and the series or parallel associations between them. The last stage connects the automatized load, selecting the cyclic or single test. Our developed control software is programmed in the National Instruments LABVIEW^™^ graphical language. The software commands the acquisition of the measured piezoelectric signal of the PDUT at the first routing stage to obtain the active piezoelectric simulation model, according to the periodical operation of the test bench. The software obtains the transient and the steady state of the energy harvesting measured voltage. The power and load regulation graphs are obtained by applying different loads. The open load voltage and the output equivalent impedance of the energy harvesting capacitor filtered rectifier circuit are computed. An example of the acquisition of four electrical signals from a PZT PDUT using the oscilloscope is presented in Figure 3a. In Figure 3b, our developed software user interface acquires channel number 1 of the piezoelectric response shown in Figure 3a.

This piezoelectric characterization system configures a new virtual instrument (VI). A virtual instrument performs the functions of the traditional measurement instruments but engineers and scientists can build automated measurement systems that suit their needs exactly instead of being conditioned and limited by standard instruments.

**Figure 4:**
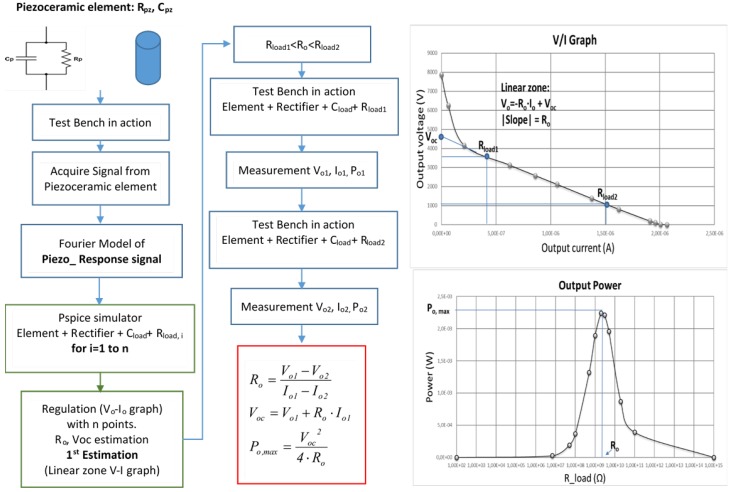
Methodology applied in the original piezoelectric characterization system.

The methodology to obtain and validate the models is presented in Figure 4. The steps that cover the process are as follows:

(1) The PDUTs are electrically characterized. Their impedance is measured with an impedance meter. The piezoelectric elements are placed in the test bench.
(2) The test bench is set in action. The piezoelectric voltage is acquired and its active Fourier model is calculated. The active Fourier model is obtained by calculating each Fourier component of the inner piezoelectric generators, taking into account the input impedance of the measurement equipment and the impedance of the PDUTs.
(3) The active Fourier model is sent to the LabVIEW^®^ PSpice-based software module. An iterative process is started. The harvesting circuit formed by a capacitor-filtered rectifier stage is simulated for n different load resistance values. The high accuracy of the active Fourier models achieves a low simulation error.
(4) The VI computes the voltage–current and power graphs. A first estimation of the open circuit voltage (*V*_oc_) and the equivalent output resistance (*R*_o_) of the harvester in the maximum power zone is obtained.
(5) The next step is to verify the accuracy of the first estimation obtained for the key parameters *V*_oc_ and *R*_o_. Analyzing the simulation results, a pair of appropriate values for the load resistance (R_load1_ and R_load2_) are chosen. These resistor values are connected in the HEH module.
(6) The test bench is set in action. The voltage, current, and power are registered for both load resistance values.
(7) The practical values of output resistance (*R*_o_), open circuit voltage (*V_oc_*), and maximum power point (Po_max_) are obtained and empirically verified.

**Figure 5:**
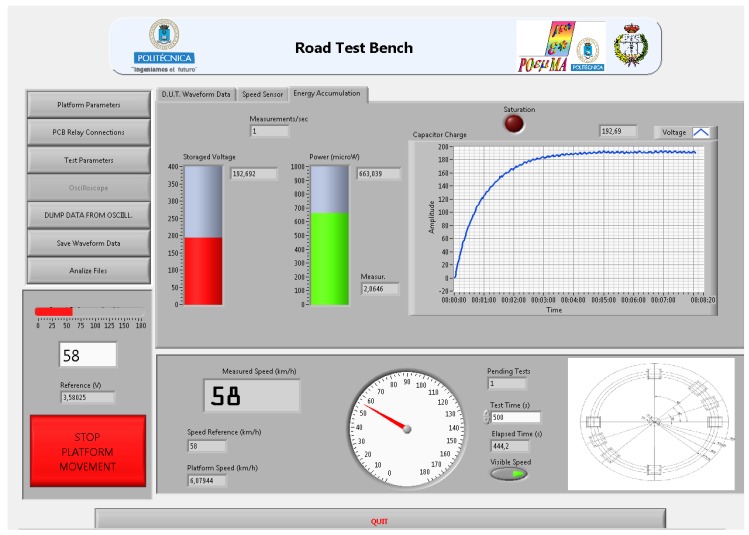
Practical test results.

The actual measurements on the harvesting electronic hardware (Figure 1) module validate the methodology. In Figure 5, the VI screen of the accumulated voltage measured in the energy harvesting module of the Test Bench is presented.

### 2.1. Piezoelectric Ceramic Material Characterization under Harvesting Conditions

**Figure 6:**
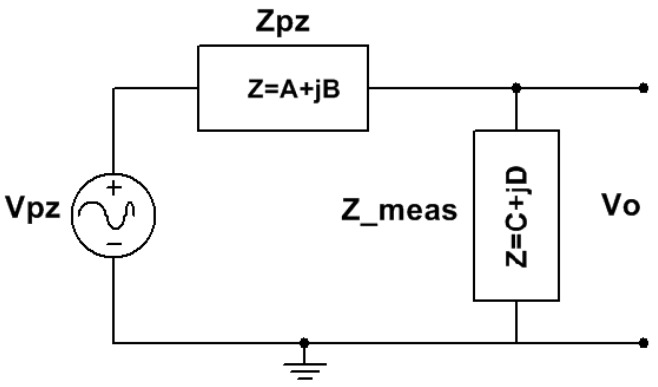
Electrical equivalent circuit needed to obtain the active piezoelectric model in energy harvesting road traffic applications.

Our methodology computes, at the first stage, an active electrical model of the piezoelectric material mechanically excited by the road traffic. The model is the series association of the impedance of the material with active inner Thévenin voltage generators. This is calculated with Fourier analysis of the measured piezoelectric voltage (Figure 3a), the equivalent input impedance of the oscilloscope, and the impedance of the piezoelectric elements. Figure 6 shows the electrical circuit needed to solve the active electrical model of the piezoelectric ceramic. The Fourier generator *V*_pz_ and the piezoelectric impedance *Z*_pz_ are the elements of the active electrical model of the piezoelectric ceramic materials. The impedance of the measurement equipment is a key factor to calculate the active Fourier electrical model that predicts its behavior in whatever energy harvesting application. In this case, the measurement oscilloscope probe (*Z*__meas_ in Figure 6) has an equivalent input impedance of 10 MΩ in parallel with a capacitance of 4 pF when it is connected to the input impedance of the oscilloscope (which is of 1 MΩ in parallel with a capacitance of 11 pF).

Equation (2) calculates the component values of the Fourier active generator *V*_pz_, when the spectrum of frequencies of the measured voltage *V*_o_ is computed by the VI. Equation (3) calculates the measurement impedance *Z*__meas_ with *C*_p_ and *R*_p_ being the capacitive and resistive values of the probe connected to the oscilloscope, respectively.

(2) $$V_{pz}\left(f_{i}\right)=\frac{V_{o}\left(f_{i}\right)}{Z_{\_meas}}\cdot \left(Z_{pz}+Z_{\_meas}\right),$$

(3) $$Z_{\_meas}=\frac{R_{p}-jR_{p}^{2}\omega_{i}C_{p}}{1+R_{p}^{2}\omega_{i}^{2}C_{p}^{2}},$$

(4) $$\omega_{i}=2\pi f_{i}.$$

Table 2 shows the values of the properties of the lead-containing and lead-free materials. The values in Table 2 show that the lead-containing material is more piezoelectric, polarizable, and lossy, as well as more compliant, than the lead-free material.

**Figure 7:**
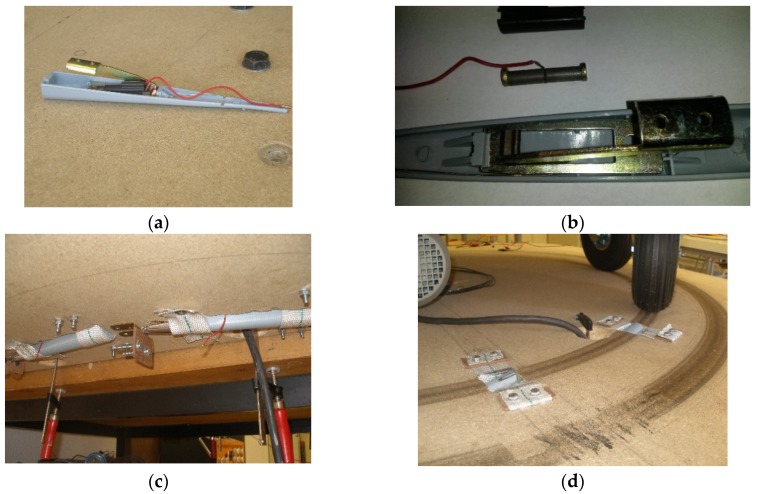
(**a**) Open view of the commercial piezoelectric housing; (**b**) exploded view of the commercial piezoelectric showing the lever mechanical amplifier and the piezoelectric material outside the holder; (**c**) bottom view of the commercial piezoelectric placement in the test bench; (**d**) top view of the PDUTs in the inner path of the road traffic simulator.

In Figure 7, the detailed housing of the PDUTs and their location in the test bench are depicted. These are two cylinders connected electrically in parallel, but mechanically in series. The piezoelectric elements are placed in a mechanically amplified (lever) holder (see Figure 7a,b in exploded view), and disposed in very shallow cavities (lever projects only 2 mm from the nonrotating platform) in diametric positions in the test bench inner path (see Figure 7c,d).

#### 2.1.1. Impedance of the PDUTs

**Figure 8:**
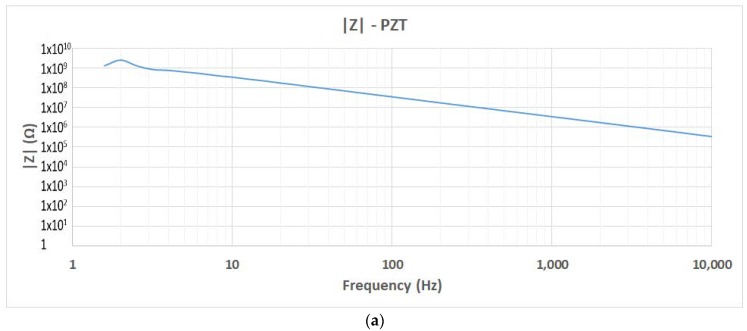

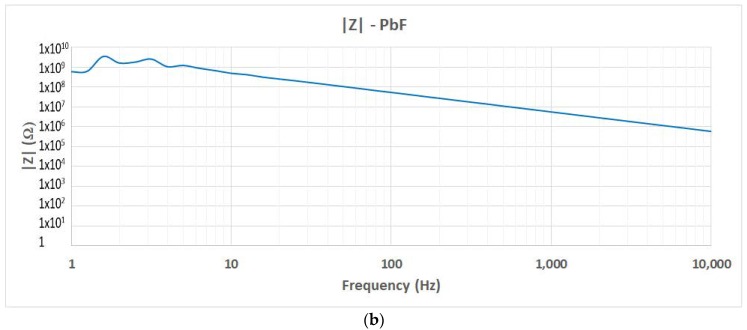
Impedance measurement of the PDUTs: (**a**) Lead-containing PZT; (**b**) lead (Pb)-free.

The impedance of the PDUTs was determined with the impedance analyzer Solartron 1260 from AMETEK Scientific Instruments. The impedance analyzer provides the real and imaginary parts of the impedance (*Z*’(*a*) and *Z*’’(*b*)). Equations (5)–(10) obtain the modulus and phase of the impedance, the admittance, the capacitance, and the resistance of the material. The results for the impedance module of PZT and PIC700 are shown in Figure 8.

(5) $${Ø}_{Z}=tan^{-1}\frac{Z^{\prime \prime }\left(b\right)}{Z^{\prime }\left(a\right)},$$

(6) $$\left|Z\right|=\sqrt{{\left(Z^{\prime }\left(a\right)\right)}^{2}+{\left(Z^{\prime \prime }\left(b\right)\right)}^{2}},$$

(7) $$\left|Y\right|=\frac{1}{\left|Z\right|},$$

(8) $${Ø}_{Y}=-{Ø}_{Z},$$

(9) $$R_{pz}=\frac{\left|Z\right|}{cos{Ø}_{Z}}=\frac{1}{\left|Y\right|cos{Ø}_{Y}},$$

(10) $$C_{pz}=\frac{-sen{Ø}_{Z}}{\left|Z\right|2\pi f}=\frac{\left|Y\right|sen{Ø}_{Y}}{2\pi f}.$$

The capacitive effect is relevant in both piezoelectric materials on the impedance of the PDUTs.

#### 2.1.2. Piezoelectrically Active Electrical Model

**Figure 9:**
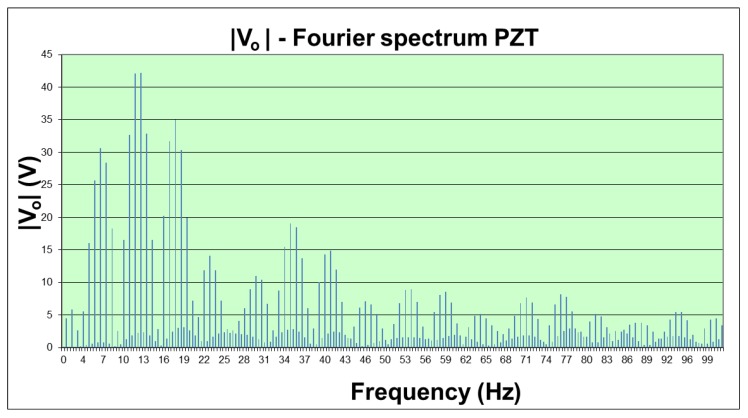
Fourier spectrum modulus of the measured voltage (*V*_o_) in the PZT ceramics.

**Figure 10:**
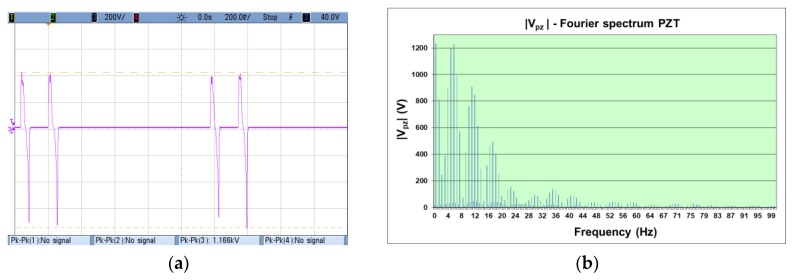

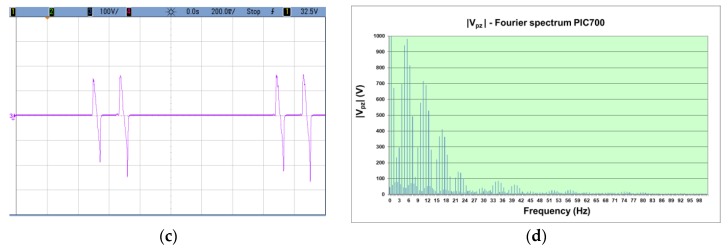
(**a**) PZT material measured voltage; (**b**) Fourier spectrum (modulus) of the active piezoelectrical generator for the PZT ceramic material; (**c**) lead-free ceramics measured voltage; (**d**) Fourier spectrum (modulus) of the active piezoelectrical generator for the PIC700 ceramic material.

The Test Bench, programmed to perform the road test at 58 km/h of simulated car speed, stresses both piezoelectric materials in the same way to the consecutive tests. The generated voltage (*V*_o_ in Figure 6) was recorded in the VI to compute their Fourier spectrum. The modulus of the PZT Fourier analysis is shown in the Figure 9. The voltage *V*_o_ measured with the oscilloscope and the modulus of the active generator from the spectral Fourier analysis, │*V*_pz_│, calculated by the VI are presented in Figure 10 for the PZT and the lead-free piezoceramics.

The amplitude of the spectral components of the measured *V*_o_ voltage is on the tens of volts range; meanwhile, the amplitude of the components in the inner active piezoelectric generator (*V*_pz_, see Figure 6) is on the order of magnitude of a thousand volts. The effect of the load impedance and the high impedance of the PDUTs explains this behavior in practical energy harvesting applications.

In energy harvesting road traffic environmental applications, the working conditions are in the very low frequency band. The frequencies of interest are always below 100 Hz because the Fourier spectral analysis of the piezoelectric response shows a bandwidth up to 100 Hz at the Test Bench maximum speed. This practical conclusion points to the main difference of this work with respect to other research works that show interest in working with piezoelectric elements in the resonance points of the material (here at ~150 kHz, see Table 2).

The recorded voltages show that lead-free piezo-ceramics generates a lower peak-to-peak voltage than the PZT material, in agreement with the values in Table 2.

Once the active electrical model is computed, it is possible to start the next stage of harvesting simulations to conclude with energy harvesting application results.

## 3. Energy Harvesting Results

**Figure 11:**
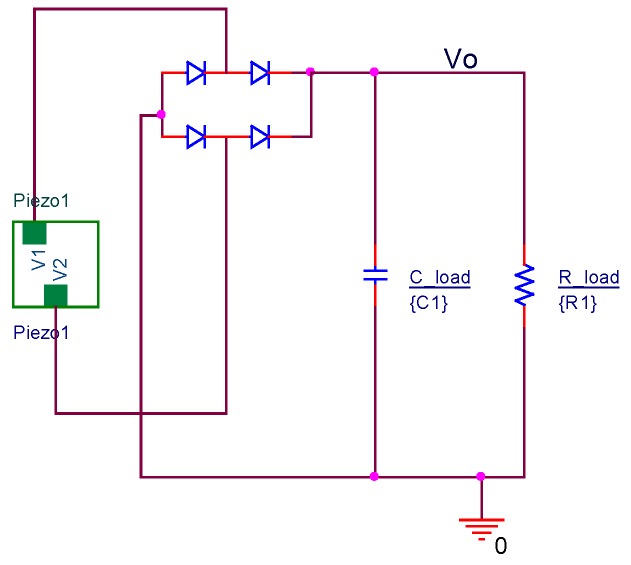
Harvesting circuit.

**Figure 12:**
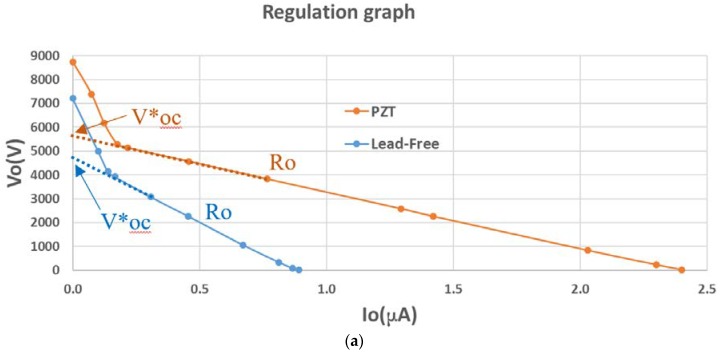

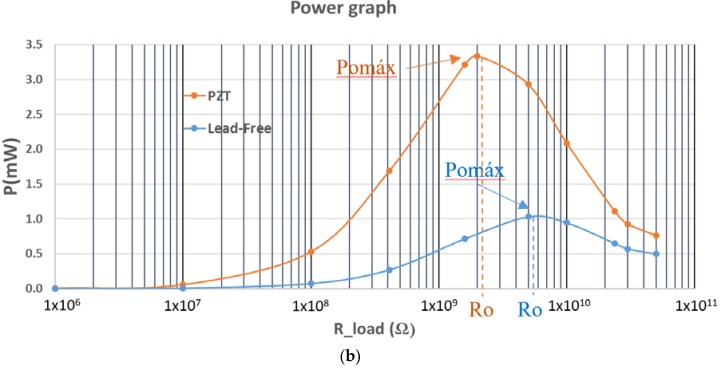
Comparative results: PZT vs. lead-free piezoceramics: (**a**) Regulation graph; (**b**) power generated graph.

The VI computes the piezoelectric active model. The model is different for each value of simulated speed. The active electrical model is exported to perform the electrical simulations in PSpice-based software connecting the piezoelectric model to the diode rectifier circuit filtered by the capacitor. The capacitor accumulates the extracted charge. The load resistance (*R*__load_ in Figure 11) is varied in successive simulations from 100 Ω (practical zone of short circuit) to 1000 GΩ (practical zone of open load) to obtain the voltage and current load graph. The practical graphic results are presented in Figure 12 for the PZT and lead-free PIC700 ceramic.

In Figure 12a, the regulation graph of voltage *V*_o_ (see Figure 11) versus load current (Io) in resistor *R*__load_ is presented for tests at 58 km/h of simulated speed using PZT and PIC700 lead-free ceramics. The parameters *R*_o_ (output resistance, calculated as the slope of the linear zone where maximum power is achieved) and V*_oc_ (open circuit voltage: Intersection of the ordinate axis with the extended line of the linear maximum power zone) are the key factors to estimate the maximum power point of the harvesting power.

The maximum extracted power point verifies Equation (11), when the *R*__load_ applied equals the output equivalent (*R*_o_) resistance of the piezoelectric harvesting circuit. The parameter *R*_o_ is previously unknown and is of significant relevance to design energy harvesting systems that achieve the maximum energetic efficiency. Our methodology calculates *R*_o_ and estimates V*_oc_ with high precision.

(11) $$Po_{max}=\frac{V_{oc}^{*2}}{4\cdot R_{o}}.$$

The practical results of the simulation stage are summarized in Table 3.

The data in Table 3 show that the impedance of the ceramic set (piezo + accumulator circuit) of maximum power delivery is approximately three times higher in the lead-free piezoelectric ceramic.

It is also observed that the deliverable power for the optimum *R*__load_ is approximately three times lower in the lead-free ceramic.

The results of the experiments verify that the tested materials are different from the point of view of electric power generation. However, the differences are not so distant. To equalize the maximum power capability, the lead-free material should be excited to provide a piezoelectric amplitude (V*_oc_) of approximately √3 times greater. This conclusion opens the way to the ecological materials in alternative energy generation.

**Figure 13:**
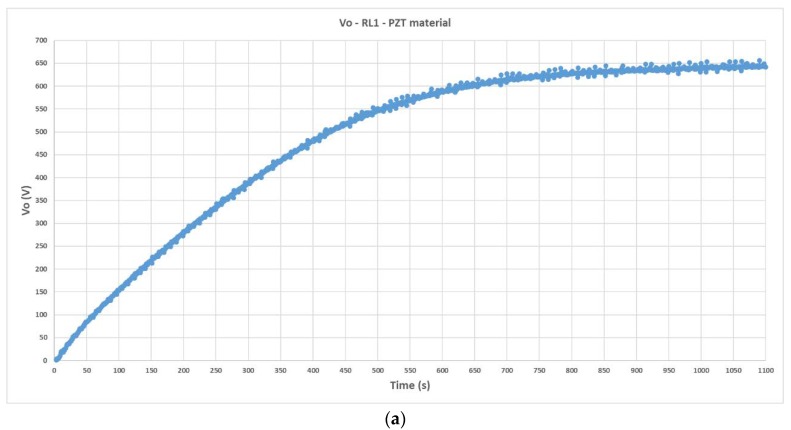

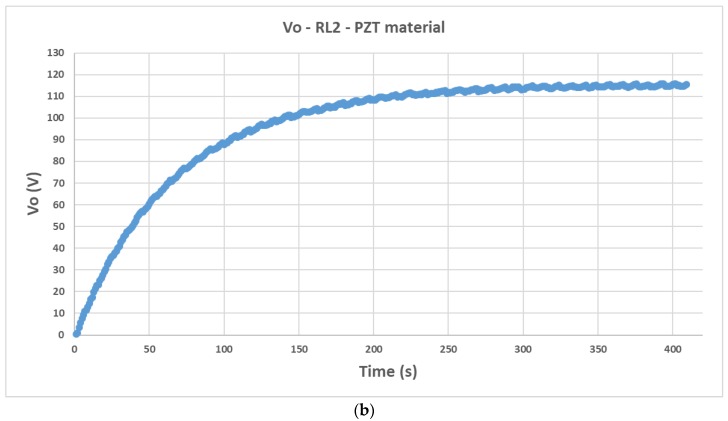
Transient response and steady state of the accumulated voltage in the capacitor (*C*__load_ = 1 μF) of the harvesting circuit when the PZT piezoelectric material is utilized in the Test Bench: (**a**) Output voltage recorded by virtual instrument (VI) when using a set of resistors of equivalent *R*__load1_ = 300 MΩ; (**b**) output voltage when *R*__load2_ = 50 MΩ.

The validation procedure stage was performed next in the Test Bench. A couple of *R*__load_ values were selected to be in the linear zone of maximum harvesting power. The practical values of the accumulated voltage *V*_o_ in the energy harvesting circuit are presented in Figure 13 for the PZT material. Table 4 calculates the practical parameter *R*_o_ and the relative error (Er) between empirically validated data and previous results from simulations.

**Figure 14:**
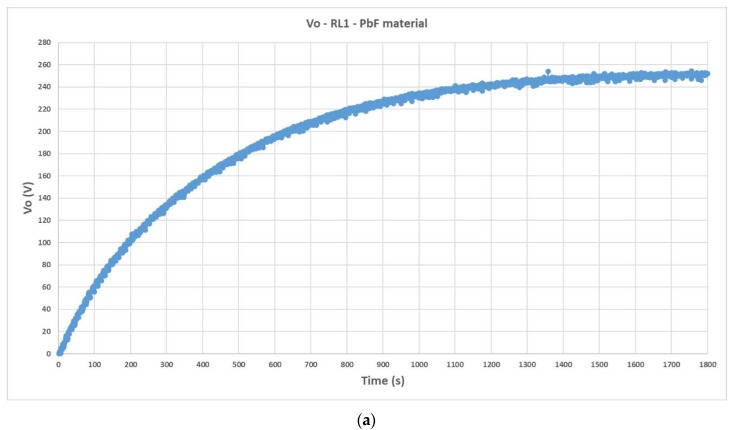

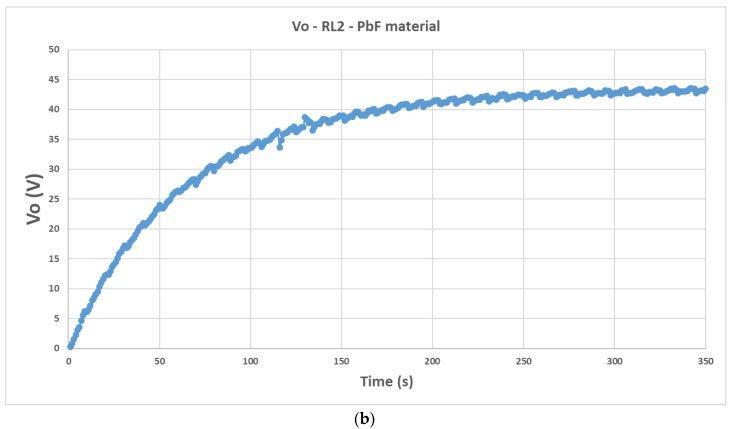
Transient response and steady state of the accumulated voltage in the capacitor (*C*__load_ = 1 μF) of the harvesting circuit when PIC700 lead-free piezoelectric material is utilized in the Test Bench: (**a**) Output voltage recorded by VI when using a set of resistors of equivalent *R*__load1_ = 300 MΩ; (**b**) output voltage when *R*__load2_ = 50 MΩ.

The measurements of the accumulated voltage in the harvesting circuit when PIC700 is utilized are presented in the Figure 14.

Table 5 presents the empirically determined *R*_o_ and the relative error achieved between previous results from simulations and test validated data.

The methodology presented allows the use of only two load resistor values to empirically verify the value of output impedance of the harvester previously determined by simulations. This value is relevant for optimizing the energy harvesting process in maximum power point algorithms.

The originality of the new instrument developed and adapted to perform road traffic tests in a laboratory environment achieves practical results with low error in the modeling characterization process of piezoelectric materials and energy harvesting systems.

The influence of the measurement equipment is considered in the development of the practical methodology exposed.

The results obtained in a single device under test can be generalized to topological associations between harvesters, as it was previously published [20]. The influence of the rate of traffic (vehicles/minute) and of peak-to-peak piezoelectric voltage on the harvested power was discussed in [21]. The topologies of associated harvesters verify the modeling process described in References [19,20,21].

Those previous results have opened the prospects of using lead-free piezoelectric materials in clean electrical energy generation.

## 4. Conclusions

The models used here to analyze and predict the energy generation of harvesters based on piezoelectric ceramics are original. With this original methodology, we were able to compare the performance in piezoelectric energy harvesting in road traffic of lead-containing (PZT) and lead-free (PIC700) piezoelectric ceramics. Classical research about energy harvesting using piezoelectric materials is based on vibrational behavior, at which the two materials present differences, particularly at resonance (see Table 2). The vibrational component in the stress applied by road traffic is not relevant in the presented analysis. The low relative error achieved between the theoretical analysis of applying the models and the practical tests with real harvesting systems is under 3% both for the lead-containing and lead-free material.

The data in Table 3 show that the impedance of the ceramic set (Piezo + accumulator circuit) of maximum power delivery is approximately three times higher in the lead-free piezoelectric ceramic. The results of the experiments verify that the tested materials are different from the point of view of electric power generation. However, the differences are not so distant. To equalize the maximum power capability, the lead-free material should be excited to provide a piezoelectric amplitude (V*_oc_) of approximately √3 times greater. This conclusion opens the way to the ecological materials in alternative clean energy generation.

## Figures and Tables

**Table 1:** Summary of road traffic energy harvesting publications. Review from 2010.

Published [Reference]	Contribution
2010 [5]	Finite elements theoretical and simulation study of the application of cymbal-type housing for piezoelectric materials. 1.2 mW generated at 20 Hz
2012 [6]	Several piezoelectric packages are studied using the finite elements technique for asphalt inlay highlighting cymbal and bridge for its efficiency in energy conversion
2015 [7]	Three encapsulation options for bridge-type housing are studied to minimize the fracture of the piezoelectric material by fatigue. It is concluded that the arch bridge is optimal for burying on asphalt. An applied pressure of 0.7 MPa generated 286 V
2016 [8]	A prototype consisting of 4, 8, or 16 piezoelectric disks sandwiched between two copper plates was assembled in-between asphalt mixtures. A uniaxial compression test was performed to measure the output power directly on a resistor
2016 [9]	Based on the Ph. D. thesis of the first author, piezoelectric degradation measurements in an USA real road installation are presented. Over 14% of the asphalt stress produced by the vehicles is transmitted to the road-embedded prototypes producing 3.106 mW of harvested power
2016 [10]	Two prototypes formed by stacked prismatic or cylindrical piezoelectric elements are tested in the laboratory. Assuming daily moderately busy USA Interstate highway traffic of 30,000 vehicles/day, the first prototype will produce 9.66 Wh per year and the second one 240.95 Wh
2016 [11]	A cymbal structure is modified in seven piezoelectric parallelized sections. In a laboratory test over a 400 kΩ resistor, 2.1 mW of power is produced
2016 [12]	An association of piezoelectric cantilevers produces 184 µW over an empirically optimized resistor of 70 kΩ. A Universal Test Machine (UTM) performs the laboratory tests
2016 [13]	Wheel tracking tests are performed assuming a continuous rate of traffic. Several recommendations are obtained to adjust the geometry and composition of the piezoelectric material in order to maximize the extracted power in response to variable speed and distance between vehicles
2017 [14]	Up to 60 PVDF layers are associated in parallel to generate 200 mW of peak power. Viability of using flexible material is shown
2017 [15]	A new structure formed by a layer of piezoelectric material embedded between two layers of conductive asphalt generates 1.2 mW in UTM tests
2018 [16]	A stacked array type of piezoelectric energy harvester is field-tested, generating a voltage between 250 and 400 V when a test vehicle is passes. The obtained piezoelectric energy lights LED signs
2018 [17]	A new prototype of 11 stacked piezoelectric elements is presented and compared to the prototype results presented in [8]. The energy output estimated per prototypes I and II was 360 and 171 Wh annually

**Table 2:** Piezoelectric (*g*_33_ and d_33_) and elastic (*s*_33_*^D^*; or *Y*_33_ = 1/*s*_33_) coefficients, dielectric permittivity and losses (*K*_33_*^T^* and tan *δ*), and electromechanical coupling factors (***k*_33_**) of the lead-containing, hard lead titanate zirconate (Navy I-type PZT; APC International, Ltd., Mackeyville, PA, USA) and lead-free, tetragonal bismuth sodium barium titanate (BNBT) (PIC700; PI Ceramic GmbH, Lederhose, Germany) commercial ceramic materials (longitudinally poled cylinders of 6 mm diameter and 15 mm length). The catalog values are shown for PZT, and PIC700 was characterized using the resonance method (*f_s_* = 148.3 kHz, *f_p_* = 160.1 kHz).

Material	*g*_33_ (10^−3^ Vm/N)	*d*_33_ (10^−12^ C/N)	*s*_33_*^D^* (10^−12^ m^2^/N)	*K*_33_ *^T^*	tan *δ* (%)	*k*_33_
PZT	26	>260	12.5	1280	0.6	>0.68
BNBT	16	98	7.5	710	0.4	0.40

**Table 3:** Parameters of the piezoelectric energy harvesting application system.

Parameter	PZT	PIC700
*R*_o_ (GΩ)	2.36	5.57
V*_oc_ (V)	5640	4800
*Po*_max_ (mW)	3.4	1.03

**Table 4:** Empirical verification of the methodology. PZT material.

Measurements	Simulations	Er %
$R_{o}=\frac{\left|V_{o1}-V_{o2}\right|}{\left|I_{o1}-I_{o2}\right|}=\frac{645-115}{\left|2.12-2.35\right|\cdot 10^{-6}}=2.30\;G\Omega$	2.36 GΩ	−2.54

**Table 5:** Empirical verification of the methodology. PIC700 lead-free material.

Measurements	Simulations	Er %
$R_{o}=\frac{\left|V_{o1}-V_{o2}\right|}{\left|I_{o1}-I_{o2}\right|}=\frac{250.7-43.6}{\left|0.835-0.873\right|\cdot 10^{-6}}=5.45\;G\Omega$	5.57 GΩ	−2.15

## References

[1] Red Eléctrica de España. https://www.ree.es/sites/default/files/11_PUBLICACIONES/Documentos/InformesSistemaElectrico/2018/inf_sis_elec_ree_2018.pdf.

[2] Villafuerte-Castrejón M.E., Morán E., Reyes-Montero A., Vivar-Ocampo R., Peña-Jiménez J.-A., Rea-López S.-O., Pardo L. (2016). Towards Lead-Free Piezoceramics: Facing a Synthesis Challenge. Materials.

[3] Calio R., Rongala U.B., Camboni D., Milazzo M., Stefanini C., de Petris G., Oddo C.M. (2014). Piezoelectric Energy Harvesting Solutions. Sensors.

[4] Abramovich H., Har-nes I. (2018). Analysis and Experimental Validation of a Piezoelectric Harvester with Enhanced Frequency Bandwidth. Materials.

[5] Zhao H., Yu J., Ling J. (2010). Finite element analysis of Cymbal piezoelectric transducers for harvesting energy from asphalt. J. Ceram. Soc. Jpn..

[6] Zhao H., Ling J., Yu J. (2012). A comparative analysis of piezoelectric transducers for harvesting energy from asphalt pavement. J. Ceram. Soc. Jpn..

[7] Zhao H., Qin L., Ling J. (2015). Test and Analysis of Bridge Transducers for Harvesting Energy from Asphalt Pavement. Int. J. Transp. Sci. Technol..

[8] Roshani H., Dessouky S., Montoya A., Papagiannakis A.T. (2016). Energy harvesting from asphalt pavement roadways vehicle-induced stresses: A feasibility study. Appl. Energy.

[9] Xiong H., Wang L. (2016). Piezoelectric energy harvester for public roadway: On-site installation and evaluation. Appl. Energy.

[10] Papagiannakis A.G., Dessouky S., Montoya A., Roshani H. (2016). Energy Harvesting from Roadways. Procedia Comput. Sci..

[11] Yesner G., Kuciej M., Safari A., Jasim A., Wang H., Maher A. Piezoelectric Energy Harvesting Using a Novel Cymbal Transducer Design. Proceedings of the 2016 Joint IEEE International Symposium on the Applications of Ferroelectrics, European Conference on Application of Polar Dielectrics, and Piezoelectric Force Microscopy Workshop (ISAF/ECAPD/PFM).

[12] Song Y., Yang C.H., Hong S.K., Hwang S.J., Kim J.H., Choi J.Y., Ryu S.K., Sung T.H. (2016). Road energy harvester designed as a macro-power source using the piezoelectric effect. Int. J. Hydrogen Energy.

[13] Chen Y., Zhang Y., Li C., Yang Q., Zheng H., Lü C. (2016). Mechanical Energy Harvesting from Road Pavements Under Vehicular Load Using Embedded Piezoelectric Elements. J. Appl. Mech..

[14] Jung I., Shin Y.-H., Kim S., Choi J., Kang C.-Y. (2017). Flexible piezoelectric polymer-based energy harvesting system for roadway applications. Appl. Energy.

[15] Guo L., Lu Q. (2017). Modeling a new energy harvesting pavement system with experimental verification. Appl. Energy.

[16] Yang H., Wang L., Zhou B., Wei Y., Zhao Q. (2018). A preliminary study on the highway piezoelectric power supply system. Int. J. Pavement Res. Technol..

[17] Roshani H., Jagtap P., Dessouky S., Montoya A., Papagiannakis A.T. (2018). Theoretical and Experimental Evaluation of Two Roadway Piezoelectric-Based Energy Harvesting Prototypes. J. Mater. Civ. Eng..

[18] DNV KEMA Energy & Sustainability (2014). Final Project Report: Assesment of Piezoelectric Materials for Roadway Energy Harvesting: Cost of Energy and Demonstration Roadmap.

[19] Vázquez Rodríguez M. (2019). Contribución al Estudio de la Generación de Energía Eléctrica a Partir de Materiales Piezoeléctricos. Ph.D. Thesis.

[20] Vázquez-Rodríguez M., Jiménez F.J., de Frutos J., Alonso D. (2016). Piezoelectric energy harvesting computer controlled test bench. Rev. Sci. Instrum..

[21] Vázquez-Rodríguez M., Jiménez F.J., de Frutos J. (2018). Virtual instrument to obtain electrical models of piezoelectric elements used in energy harvesting. Adv. Appl. Ceram..

